# Delaying Intraoral Radiographs during the COVID-19 Pandemic: A Conundrum

**DOI:** 10.1155/2022/8432856

**Published:** 2022-01-12

**Authors:** Harneet Kaur, Harshita Gupta, Himanshu Dadlani, Gulsheen Kaur Kochhar, Gurkeerat Singh, Ritasha Bhasin, Anuraj Singh Kochhar, Mohammad Khursheed Alam

**Affiliations:** ^1^Department of Orthodontics and Dentofacial Orthopaedics, Faculty of Dentistry, Jamia Milia Islamia, New Delhi 110025, India; ^2^Department of Orthodontics and Dentofacial Orthopaedics, Sudha Rustagi College of Dental Sciences and Research, Faridabad, Haryana 121002, India; ^3^Department of Periodontology, Kalka Dental College and Hospital, Meerut 250006, India; ^4^Department of Pediatric Dentistry, Swami Devi Dyal Hospital and Dental College, Panchkula, Haryana 134118, India; ^5^Faculty of Dentistry, University of Toronto, M5G1G6, ON, Canada; ^6^Orthodontics and Dentofacial Orthopaedics, Faculty of Dentistry, University of Toronto, M5G1G6, Toronto, Canada; ^7^Orthodontic Division, Preventive Dentistry Department, College of Dentistry, Jouf University, Sakaka 72345, Saudi Arabia; ^8^Department of Dental Research Cell, Saveetha Dental College and Hospitals, Saveetha Institute of Medical and Technical Sciences, Chennai, India; ^9^Department of Public Health, Faculty of Allied Health Sciences, Daffodil lnternational University, Dhaka, Bangladesh

## Abstract

**Background:**

The COVID-19 pandemic has made dentists very assiduous about cross-infection during dental treatment, thereby delaying dental radiographs for treatment. However, patients needing dental emergency treatment in the ongoing pandemic require relevant intra/extraoral dental radiography for adequate diagnosis and treatment planning.

**Methods:**

This article is aimed at adding to the hot debate: Is delay for intraoral radiographs justified or a possible proxy? As a narrative review, it provides an insight into the reasons for delaying intra-oral dental radiographs during in the pandemic and options of the nontraditional radiographic techniques available until the pandemic subsides. *Discussion and Conclusion*. Cross-contamination concerns through respiratory droplets grow while using intraoral film holders that stimulate gag reflex, coughing, saliva secretion, and if proper disinfection protocols are not applied. Since the patients' acquiring emergency dental treatment cannot be neglected, the return-to-work guidelines by the health regulatory bodies urge to prioritize extraoral radiographic imaging techniques to curb the infection, offering the best diagnostic efficacy. The dental professionals can consider cone-beam computed tomography (CBCT) scans and sectional dental panoramic radiographs (SDPRs), followed by a risk assessment for COVID-19, a safer modality in reducing cross-contamination and assuring an innocuous environment for both patient and coworkers.

## 1. Introduction

The COVID-19 pandemic has been a multidimensional global crisis, increasing the need for optimal healthcare resources [1]. Most of the elective procedures in dentistry have been suspended or delayed [2, 3]. In this predicament, dental radiography, the backbone for decision-making for diagnosis and treatment regimens in dentistry, has also been majorly affected [4, 5]. The COVID-19-based infection control protocols in dentistry have also changed the imaging algorithm based on the exposure risk to patients and healthcare workers. This revisit adds to the hot debate—whether delay for intraoral radiographs is justified?

## 2. Methodology

### 2.1. Where Precisely the Problem Begins?

According to the WHO/ADA categorization of dental procedures for COVID safety, we can also grade the dental radiographs as per the need based on the same categorization. (Table 1) [6, 7]. This categorization clears the pavement to the enigma that IOPA or bitewing radiographs are the most routinely used dental radiographs and need a proxy in emergencies [8]. Aerosol-generated transmission of COVID-19 means the person-to-person transmission of the deadly virus through respiratory droplets [9, 10]. Hence, the concerns of aerosol generation during intraoral radiography by coughing or gagging induced from film holders are valid in COVID-19 infection control guidance. This includes using appropriate personal protective equipment by dental practitioners and radiography staff per World Health Organization 2020b protocol [11].

Gag is amongst the common problems associated with intraoral radiographs. The overall frequency of gagging during intraoral radiography differs significantly when the radiographs are taken by experienced radiographical workers [12]. Also, it cannot be denied that gag due to positioning of intraoral receptors in all sites is most typical in the maxillary molar area [13]. Thus, the prescription of intraoral radiography for selective criteria is required to be published in association with the most recent guidelines about COVID-19 pandemic infection prevention protocol [14]. Till then, an interim guidance for reducing aerosol generation due to intraoral radiography during the COVID-19 crisis should be considered [15].

### 2.2. The Alternatives to Intraoral Radiography

Sectional dental panoramic radiographs (SDPRs) and cone-beam computed tomography (CBCT) scans have been prioritized over intraoral radiography due to the ongoing pandemic [16]. A particular crisis like unstable maxillofacial fractures, a diffuse soft-tissue bacterial infection that can compromise the patient's airway or uncontrolled postoperative bleeding, can be attended with the multidisciplinary approach to substitute IOPA like CBCT, panoramic radiography [17].

Only interventions for dental emergencies are managed in dedicated clinics, following a formal risk assessment for COVID-19. In these interventional procedures, relevant imaging and dental intraoral radiography have been advantageous since traditional times [11]. In routine diagnosis-based imaging like oral examination, aesthetic dental procedures, restorative treatment, extraction of asymptomatic teeth, dental cleaning, replacement of missing tooth/teeth with fixed or removable prosthesis, and/or routine orthodontic visits, intraoral radiography can be advantageous in non-COVID patients [2]. Some clinicians may prefer SDPR and CBCT equipment over intraoral radiography instead of preventing aerosol productions by repeated imaging exposures for IOPAs.

*Now the question is if intraoral radiography offers the best diagnostic efficacy*. The answer is, probably, not. SDPRs are reasonable in an emergency setting, eliminating the probable gag due to intolerable intraoral films [18]. However, CBCT cannot be an alternative to intraoral radiography—mainly because of the higher radiation dose exposure that is not encouraged in routine procedures where bare minimum 2D imaging stands diagnostically acceptable [19].

## 3. Discussion

The SARS-CoV-2 virus is unfathomable and can persist for extended periods in aerosol and is potentially contagious via intraoral radiography through gagging and coughing [20]. During this unusual pandemic straw-hat, the goal is to keep dental radiography simple to minimize staff-to-patient contact with qualitative diagnostic radiographs. Various healthcare international bodies have advocated sectional (SDPRs) or full-width dental panoramic radiography (OPG) or CBCT to be the first line of imaging [21, 22], adequate for managing patients in acute settings considering emergency interventional strategy-based dental treatment.

It may be pretentious to mention that intraoral radiographs, if required, can be taken with caution due to the potential of patient aerosol production from coughing, gagging, retching, or vomiting by film holders (even in COVID-19 negative cases) [22, 23]. However, various constraints were imposed on dental practice during the COVID-19 era, limiting the unrestricted use of intraoral radiography and encouraging different technologies, like SDPRs and CBCT. This will allow dental practitioners to accomplish corresponding intraoral radiography goals without aerosol generation [24].

**Figure 1:**
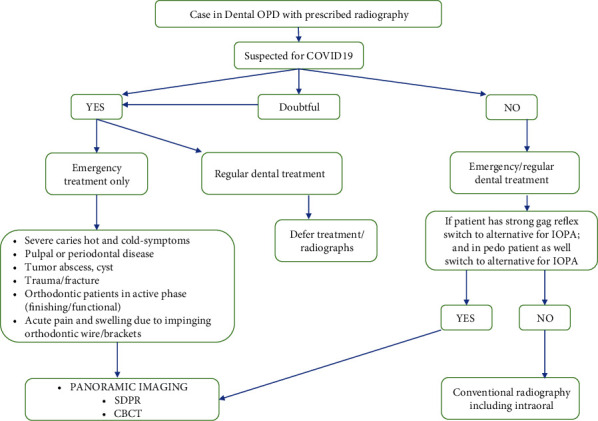
A summary algorithm for dental radiology for COVID and non-COVID patients.

Under cautions of the COVID-19 pandemic, we have proposed a broad overview summarized in an algorithm that compares the strengths, limitations, and radiation burdens of intraoral radiography, SDPR, and CBCT (Figure 1). It is significant to mention that intraoral radiography in dentistry is compromised by COVID-19 attributing to review other radiographic technologies [25]; this will emphasize thorough clinical examination and history taking. In intraoral radiography, some literature-based evidence advocates bitewings that detect mainly proximal caries, swellings, or tumours [26]. These can be solely revealed via diagnostic observations, and the clinical management decisions for a noncavitated lesion to treat nonoperatively need to be handled in COVID negative cases. In a cavitated lesion, intraoperative interventions can rely on a visual-tactile method through superior strategy, resulting in appropriate clinical management [27]. A multidisciplinary approach—an oral radiologist, working closely with the maxillofacial surgeon/oncologist/oral pathologist—is essential to maximize the chances for definitive diagnosis and minimize potential complications in biopsy procedures for tumour cases. Due to high soft-tissue resolution, CBCT offers a noninvasive delineation of soft tissues with high accuracy [28]. Still, the antiquity for bitewing and caries studies being four decades older is present, along with a crucial solution offered by CBCT for former [29]. Since CBCT is highly accurate for displaying proximal caries [30], using an appropriate imaging algorithm for different clinical conditions is essential to attain these objectives.

## 4. Conclusion

The delay for intraoral radiographs seems to be justified in the COVID-19 crisis. It is undeniable that radiological workflow protocols and policies applicable to various emergency clinical conditions/investigations must be readdressed. This will help to accomplish an imaging investigation making it vital to have an interdisciplinary dental streamed interaction. The conundrum justifies the role of proxy to intraoral radiography- cone-beam computed tomography (CBCT) scans and sectional dental panoramic radiographs (SDPRs)- in the new infection control protocol until the pandemic subsides.

## Figures and Tables

**Table 1:** Grading dental radiographs according to their situational needs.

Grade for dental radiographs	Clinical categorization	Situation	Radiographs that may be required for diagnosis and/or management
Grade I (critical)	Emergency clinical situation	(i) Unstable maxillofacial fractures	CBCT, OPG, lateral cephalogram, SMV, IOPA
		(ii) Diffuse soft-tissue bacterial infection with intraoral or extraoral swelling that compromises patient airway	CBCT, OPG, IOPA, bitewing radiograph
		(iii) Uncontrolled postoperative bleeding	CBCT/contrast-enhanced CBCT
Grade II (emergency)	The emergency clinical situation can be managed without or minimal aerosol generation	(i) Pulpal inflammation that requires tooth extraction and associated severe pain	IOPA, bitewing
		(ii) Avulsion/luxation	IOPA, occlusal radiograph
		(iii) Dry socket	None/IOPA
		(iv) Pericoronitis	IOPA, OPG, CBCT
		(v) Defective fixed orthodontic appliance causing soft-tissue laceration	IOPA, OPG
		(vi) Localized dental/periodontal abscess	IOPA, bitewing, OPG, occlusal radiograph
		(vii) Debonded fixed prosthesis cleaning and temporary cementation	IOPA, OPG
		(viii) Fractured or defective fixed prosthesis causing soft tissue injury	IOPA, OPG, bitewing, CBCT
		(ix) Removable dentures adjustments for radiation/oncology patients	-
		(x) Acute periodontal disease	IOPA, bitewing, OPG, CBCT
Grade III (routine)	Routine dental procedures that require considerations	(i) Asymptomatic fractured or defective restoration	IOPA, bitewing, occlusal radiographs
		(ii) Chronic periodontal disease	IOPA, OPG, CBCT
Grade IV (elective)	Elective procedures that can be awaited till discretion	(i) Annual oral examinations	IOPA, OPG, lateral cephalograms
		(ii) Orthodontic diagnosis	OPG, lateral cephalograms, PA cephalogram, occlusal radiographs, CBCT
		(iii) Recall visits	IOPA, OPG
		(iv) Esthetic dental procedures	IOPA, bitewing
		(v) Restorative treatment or extraction of asymptomatic teeth	IOPA, bitewing, OPG
		(vi) Routine dental cleaning and preventive therapies	IOPA, bitewing, OPG
		(vii) Replacement of missing tooth/teeth with a fixed or removable prosthesis	IOPA, OPG, CBCT
		(viii) Orthodontic records and follow-ups	OPG, lateral cephalogram, IOPA, occlusal radiographs, CBCT

## Data Availability

All data are available within the manuscript.

## Abbreviations

COVID-19: Coronavirus disease 2019
WHO: World Health Organization
ADA: American Dental Association
IOPA: Intraoral periapical radiograph
OPG: Orthopantomogram/panoramic radiograph
SDPR: Sectional dental panoramic radiographs
SMV: Submento-vertex X-ray
CBCT: Cone-beam computed tomography
PA: Posteroanterior.
